# Regulation of HDAC6 Catalytic Activity in Cancer: The Role of Post-Translational Modifications and Protein–Protein Interactions

**DOI:** 10.3390/ijms26031274

**Published:** 2025-02-01

**Authors:** Leen Asaad, Benjamin Pepperrell, Emma McErlean, Fiona Furlong

**Affiliations:** 1 School of Pharmacy, Queen’s University Belfast, Belfast BT7 1NN, UK; 2 Department of Pharmacology and Biomedical Sciences, Faculty of Pharmacy and Medical Sciences, University of Petra, Amman 11196, Jordan

**Keywords:** HDAC6, cancer, post-translational modification

## Abstract

Histone deacetylase 6 (HDAC6) is a large multidomain protein that deacetylates lysine residues on cytoplasmic proteins, influencing numerous cellular processes. Both the catalytic and noncatalytic functions of HDAC6 have been implicated in cancer development and progression. Over a decade of research on catalytic domain inhibitors has shown that these drugs are well tolerated, exhibit anticancer activity, and can alleviate chemotherapy-induced peripheral neuropathies. However, their effectiveness in treating solid tumours remains uncertain. HDAC6 activity is regulated by protein–protein interactions and post-translational modifications, which may allosterically influence its catalytic domains. As a result, effective inhibition of HDAC6 in cancer using small molecule inhibitors requires a more sophisticated understanding of its role within tumour cells, including whether its expression correlates with deacetylase activity. A comprehensive understanding of cancer-specific HDAC6 expression, functional activity, and activation states will be critical for refining the use of HDAC6 inhibitors in cancer therapy.

## 1. Introduction

Histone acetylation and deacetylation are important epigenetic mechanisms involved in gene regulation and abnormalities in this process are associated with tumorigenesis, progression, and chemo-resistance [1]. Histone acetyltransferase (HAT) enzymes add acetyl groups to lysine residues of histone proteins, while histone deacetylases (HDACs) remove them. Both enzymes belong to protein superfamilies and regulate the acetylation levels of various nuclear and cytoplasmic proteins, a process essential for maintaining normal cellular function [1]. There are 18 human HDAC proteins, of which 11 are known as the “classical” HDACs, grouped into two families and four classes based on homology and similarity to yeast factors [2]. Histone deacetylase 6 (HDAC6) is described as a Class IIb HDAC protein and one of six cytoplasmic HDAC proteins. It is the largest HDAC enzyme and uniquely consists of two catalytic deacetylase domains (CD1 and CD2) [3]. Both domains are composed of similar amino acid sequences, conformation, and architectural structure. However, the CD2 domain of HDAC6 is considered to be the main catalytic site and bears a wider substrate specificity compared to the CD1 site [4]. HDAC6 also possesses additional functional domains that further contribute to its unique properties and functions. The dynein motor binding region (DMB) of HDAC6 binds to dynein motor proteins and links the CD1 and CD2 domains together. The cytoplasmic localisation of HDAC6 is facilitated by a nuclear export signal (NES) that prevents its accumulation in the nucleus and an SE14 Ser-Glu-containing tetrapeptide region serves as a stable cytoplasmic anchor. The C-terminal zinc finger ubiquitin binding domain (ZnF-UBP) enables HDAC6 to bind mono- and poly-ubiquitinated proteins [4].

The functions of HDAC6 are intrinsically linked to its distinct protein domains, with its catalytic domain primarily responsible for deacetylating cytoplasmic proteins. Key HDAC6 substrates include α-tubulin, cortactin, and the heat shock protein 90 (HSP90) and exemplify the main physiological responses of HDAC6 [3]. Alpha-tubulin is an essential microtubule protein that is acetylated at Lys40 residue. Its deacetylation by HDAC6 is critical for maintaining microtubule stability and integrity [5]. Thus, the deacetylation of α-tubulin by HDAC6 plays a crucial role in maintaining microtubular integrity and stability, which are essential for cell division, cell motility, polarity, subcellular transport, survival, adhesion, and immune and inflammatory responses [5,6]. Meanwhile, the deacetylation of cortactin by HDAC6 connects HDAC6 activity to actin cytoskeleton dynamics and is a potential link between microtubule dynamics and the regulation of actin filament polymerisation [7,8]. The deacetylation of HSP90 by HDAC6 highlights the critical role of HDAC6 in regulating the chaperone function of heat shock proteins, which maintain the levels of pro-survival and pro-growth proteins in cells [6]. Research continues to uncover new HDAC6 substrates that have significantly broadened the functional scope of this enzyme (Table 1); however, HDAC6 also possesses catalytic-independent functions [9]. The catalytic-independent functions of HDAC6 connect acetylation and ubiquitination pathways, which are pivotal components of the pathways involved in protein aggregate accumulation, proteasomal degradation of misfolded proteins, and autophagy [10,11]. The ubiquitin binding function of HDAC6 is mediated by the ZN-UBP domain and is a critical step that regulates a chain of events involved in the clearance of cytotoxic proteins. When HDAC6 senses the accumulated levels of ubiquitinated protein aggregates, Heat Shock Factor 1 (HSF1) is released from a repressive complex with HSP90, freeing it to induce the expression of major chaperone proteins [10]. The deacetylation of the heat shock cognate protein 70 (Hsc70) and DNAJ homolog subfamily A member 1 (DNAJA1) by HDAC6 were shown to enhance Hsc70 and DNAJA1 binding, thereby promoting their chaperone function [12]. Thus, HDAC6 protein complexes provide an essential protective mechanism against cellular stress [10,11]. The DMB domain of HDAC6 acts as a molecular bridge between dynein motor proteins and ubiquitinated proteins supporting the cell to clear and recycle cellular proteins. Following tubulin acetylation by HDAC6, the molecular motors, dynein and kinesin-1, are recruited to microtubules, where the ubiquitinated protein cargoes bound to the Zn-UPB of HDAC6 are ferried along microtubules. Once at the microtubule-organising centre (MTOC), the cargo becomes fused with lysosomes and the protein aggregates are removed by lysosomal degradation and autophagy [10]. The dysregulation of cellular responses involving HDAC6 is a major underlying mechanism contributing to human disease and has identified HDAC6 as a key drug target. This review will focus on the role of HDAC6 in cancer, exploring the post-translational regulation of its catalytic domain by phosphorylation, acetylation, and protein–protein interactions. These regulatory mechanisms are rarely considered in research on HDAC6 inhibition.

## 2. Expression and Function of HDAC6 in Cancer

### 2.1. HDAC6 Expression in Tumours and Cancer Cells

Overexpression of HDAC6 has been reported in various tumour types, making it a promising target for anticancer therapy (22). HDAC6 expression is upregulated in low-grade and high-grade ovarian cancers [8,28,29], oral squamous cell carcinoma [30], hepatocellular carcinoma [31], and acute myeloid leukaemia (AML) [32] and is associated with increased chemotherapy resistance in acute lymphoblastic leukaemia [33]. In ovarian cancer and hepatocellular carcinoma, high HDAC6 is associated with advanced clinical stage, higher number of tumours, vascular and intrahepatic invasion, and metastasis, which are related to poor prognosis, respectively [31]. Therefore, HDAC6 may be correlated to the tumour aggressiveness and progression [8,30,31]. In contrast, Ali et al., 2020 showed that HDAC6 overexpression in high-grade serous ovarian cancer (HGSOC) associated with more favourable disease prognosis [29]. The low HDAC6 levels in HGSOC were associated with an increased risk of death compared to high HDAC6 patients. However, the overexpression of HDAC6 in HGSOC cell lines is associated with higher migration and proliferation rates in which low HDAC6 expression in ovarian cancer cells may represent a hard-to-treat tumour phenotype associated with increased senescence [29]. Similarly, in breast cancer patients, higher levels of HDAC6 are associated with smaller tumour size and low histological grade, in which the overexpression of HDAC6 is predominantly found in progesterone- and oestrogen-positive (ER+ve) tumours [34]. In MCF-7 ER+ve breast cancer cells, oestrogen levels affected HDAC6 localisation in the nucleus, leading to the deacetylation of survivin. This caused its nuclear export and inhibited caspase-dependent apoptosis [23]. Therefore, tamoxifen, an oestrogen receptor (ER) antagonist, sensitised the cells to apoptosis and death [23], in which high HDAC6 in breast cancer is considered to be a better prognostic factor, which correlated with higher disease-free survival rate and better endocrine therapy response [35]. HDAC6 overexpression is also described in some oestrogen-negative breast cancers [36]. However, Rosik et al. reported that urothelial cancer showed moderate expression of HDAC6, which was not associated with tumour growth or survival [37]. Overexpressed HDAC6 in oesophageal squamous cell carcinoma (ESCC) is associated with poor prognosis [38]. Furthermore, HDAC6 overexpression was reported to be more pronounced in metastatic lymph nodes (MLNs) of ESCC and, to a lesser extent, in the primary tumour [39,40]. Lower HDAC6 expression in MLNs compared to primary tumours is considered an independent prognostic marker associated with better survival rate for oesophageal cancer patients [39,40]. Generally, HDAC6 is also found to be an oncogene modulator in gastrointestinal cancers [40]. It is also overexpressed in pancreatic cancers but does not affect pancreatic cancer proliferation and migration [41]. Other studies reported no significant difference between HDAC6 expression in pancreatic cancer cell lines compared to normal human pancreatic ductal epithelial cells [42]. The analysis of 33 tumours using multiple data sources from The Cancer Genome Atlas (TCGA) and the NCI-60 drug screening database revealed that most tumours exhibited lower levels of HDAC6 and HDAC10 compared to normal tissues [43]. In inflammatory breast cancer (IBC), HDAC6 is not overexpressed but its activity is significantly higher in IBC cells compared to non-IBC cells, indicating a complex relationship between mRNA expression, translation, and protein function [44]. Furthermore, HDAC6 mRNA consists of ~23 splicing sites and 33 transcript splice variants of HDAC6 have been reported in the human gene, highlighting the functional versatility and regulatory complexity associated with its expression [45]. A novel truncated mRNA transcript for HDAC6, Hhdac6p114 variant, which was described in lung and breast cancer cells lines, is devoid of the HDAC6 nuclear export signal. Hhdac6p114 was found to be important in mediating transforming growth factor beta 1 (TGF-β1) gene activation and the regulation of epithelial–mesenchymal transition (EMT) signalling [46]. Epigenetic regulation of HDAC6 expression has been described in male breast cancer where the HDAC6 gene is significantly hypomethylated [47]. The regulation of HDAC6 by microRNAs (miRs) is also evident in various cancers, where its expression is anticorrelated with miR-26, miR-433, miR-221, miR-206, miR-22, and miR-548 in various cancers [48]. While the implications for patient prognosis are unclear from HDAC6 tumour expression, its overexpression and inhibition in disease models of cancer strongly support its involvement in cancer development and progression.

### 2.2. The Cancerous Role of HDAC6

The fundamental role of HDAC6 in the development of tumours, the transformation of normal cells into malignant ones, and the survival of cancer cells has been extensively described [3,7,8,13,17,35,41,44,49,50,51,52,53,54,55,56,57,58,59,60,61]. The requirement for HDAC6 expression in malignant cell growth was confirmed when HDAC6-null mice and HDAC6-deficient fibroblasts were shown to be more resistant to tumour formation. Furthermore, the loss of HDAC6 caused tumour cells to be less responsive to the oncogenic RAS pathway and activation of its signalling cascade [62]. The oncogenic role of HDAC6 is further supported by its regulation of microtubule dynamic [13]. During cell division, HDAC6 inhibition disrupts the formation of the mitotic spindle, although the underling mechanism is not fully understood. Microtubule-independent functions of HDAC6 contribute to cancer cell proliferation through mechanisms involving the deacetylation of substrates that are involved in growth factor signalling and regulation of the cell cycle. In contrast, decreased α-tubulin acetylation has been strongly implicated in causing microtubule instability in cancer metastasis. Increased cortactin deacetylation by HDAC6 overexpression in cancer cells provides further evidence for the role of HDAC6 in promoting cancer cell migration and invasion. The hyperacetylation of cortactin in hepatocellular cells following treatment with HDAC6 inhibitors prevents it from interacting with F-actin and blocks actin binding assembly with a marked reduction in the migration and invasion capacity of these cells [31]. Cortactin may be considered a significant predictive marker of tumour progression and reduced survival in prostate cancer, and its regulation by HDAC6 may also contribute to its role in poorer patient outcomes [63]. The regulation of microtubule dynamics by HDAC6 is also implicated in lysosomal trafficking and degradation of epidermal growth factor receptor (EGFR). Upregulated EGFR activity is a major drug target for the treatment of solid tumours and the microtubule-dependent transport of EGFR endosomal vesicles by α-tubulin acetylation links HDAC6 to the pathogenesis of prostate, pancreatic, and lung cancer cells [13].

In addition to its critical role in regulating microtubule dynamics and promoting cancer cell migration and invasion, HDAC6 influences other key oncogenic processes, including DNA damage response, apoptosis, and interactions with tumour suppressor pathways [48]. In colorectal cancer, HDAC6 expression is negatively correlated with the transcriptional activity of P53 and the deacetylation of P53 by HDAC6 leads to its inactivation [64]. The radiosensitivity of glioma stem cells is promoted when the DNA damage repair protein, checkpoint kinase 1 (CHK1), is degraded following HDAC6 inhibition [65]. MutL homolog 1 (MHL1) and the mismatch repair proteins (MMR), MSH2 and MSH6, interact and are deacetylated by HDAC6, resulting in decreased sensitivity to DNA-damaging agents in glioblastoma [66]. The induction of apoptosis alone or in combination with DNA damage is attributable to HDAC6 inhibition in several cancers, including renal cell carcinoma [67], castration-resistant prostate cancer, and oral squamous cell carcinoma [68,69] resulting from the inhibition of the antiapoptotic role of HDAC6, which helps cells survive. Binding and the sequestration of the proapoptotic protein BAX by Ku70 is modulated by HDAC6 deacetylation of Ku70 and survivin deacetylation by HDAC6 prevents it from translocating to the cytoplasm, where it is involved in the inactivation of caspases. The anti-multiple myeloma effect of HDAC6 inhibition in combinations with proteosome inhibitors is a well-established HDAC6-targeting drug combination resulting in cell death by inactivating the cell responses that deal with cytotoxic proteins [70].

More recent research has described the functional role of HDAC6 in the regulation of energy metabolism of cancer cells and cancer immune responses. Mass spectroscopy (MS) identified the HDAC6 acetylome of triple-negative breast (TNBC) cells, revealing approximately eighty-three intracellular proteins displaying increased acetylation due to catalytic inhibition of HDAC6. Specific signalling pathway analysis showed significant alterations in metabolic cellular processes resulting from HDAC6 inhibition, in which reduced aldolase and gyceraldehyde-3-phosphate dehydrogenase (GAPDH) activity was linked with increased acetylation and decreased glycolysis in TNBC cells [71]. HDAC6 has recently emerged as a major target in cancer immunotherapy because of its role in regulating the transcriptional activity of STAT3 and expression of a range of immune signalling proteins, most notably, cell death protein-1 (PD-1) and programmed death ligand 1 (PD-L1). Binding between PD-1 and PD-L1 dampens the immune response against the developing tumour, allowing tumour cells to evade immune destruction. Immunotherapy targets this response and switches off the antitumour immune blockade. Research has shown that HDAC6 knockdown or inhibition switches off the transcriptional activity of STAT3 by a mechanism that does not involve decreased STAT3 acetylation. HDAC6 and STAT3 were shown to interact and bind to DNA, resulting in a transcriptional response that drives immune tolerance in antigen-presenting cells (APCs) [72]. The interaction between HDAC6 and STAT3 has been confirmed in melanoma cells and osteosarcoma cells, although the exact mechanism is not clear [73,74]. In general, HDAC6 inhibition has been shown to decrease STAT3 transcriptional activity of the immune checkpoint proteins [75]. Moreover, downregulation of the expression of immune-suppressive proteins and PD-L1 leads to a sensitisation of tumours to immune checkpoint inhibitor treatment [76].

### 2.3. HDAC6 as a Target in Cancer

Over the past decade, a library of HDAC inhibitors has been developed, with several *pan*-HDAC inhibitors advancing to the clinic for the treatment of cancer. The most commonly used examples include vorinostat (SAHA), belinostat, panobinostat, chidamide (tucidinostat), and romidepsin [51]. The common HDAC inhibitors typically conform to a pharmacophore model consisting of three motifs: an aromatic amide “CAP” group for surface recognition, a zinc-binding group (ZBG), and a linker that bridges the ZBG and CAP. The nonselective nature of pan-HDAC inhibitors, which causes dose-limiting toxicities, along with the increasing evidence supporting the specific role of HDAC6 in cancer led to the development of isoform-selective inhibitors. While no selective HDAC6 inhibitors are utilised clinically, seven such inhibitors are undergoing clinical trials. These include ricolinostat (ACY-1215), citarinostat (ACY-241), KA2507, and AVS100 (SS-208), which are being tested for various cancers like lymphoid malignancies in relapse, metastatic breast cancer, melanoma, non-small cell lung cancer, and solid tumours (Table 2). Ricolinostat is at the most advanced stage of development and exemplifies the promising results of selective HDAC6 inhibition regarding the safety, tolerability, and efficacy in treating blood cancers such as leukaemia, multiple myeloma, and lymphoma [77,78,79,80,81]. Results from existing phase I and II clinical trials of ricolinostat showed a maximum tolerated dose of 160 mg daily dosing for ricolinostat used in combination with bortezomib or lenalidomide/pomalidomide and dexamethasone for the treatment of multiple myeloma [80]. These clinical trials showed a higher therapy response, the least adverse effects, and better disease stabilisation compared to standard treatment [70]. Using ricolinostat as a single agent demonstrated a favourable efficacy and safety profile in patients with relapsed or refractory lymphoid malignancy [82]. These trials also showed a potentially meaningful impact of ricolinostat in preventing taxane-induced neuropathy [83,84]. The efficacy of ricolinostat has also been tested in solid tumours of the breast, gynaecological cancers, and lung and biliary tract cancers and showed synergetic anticancer properties in combination with several chemotherapies, proteasomal inhibitors, and immunomodulatory drugs [82,85,86]. Selective HDAC6 inhibitors, despite their development, have shown limited efficacy in trials as a monotherapy. Although combination therapies involving HDAC6 inhibitors have demonstrated better efficacy across various cancer types, these therapies are not without challenges, including producing overlapping toxicities. Consequently, the rational design of novel, HDAC6-based multi-target agents is under development as a way to maximise the effects of HDAC6 inhibition for treating cancer [87]. Of note, there is a lack of patient studies supporting widespread use of HDAC6 inhibition in solid tumours where the limited number of clinical trials and the small number of treated patients have largely contributed to inconclusive clinical results [85,86,88,89,90]. Furthermore, what makes a cancer cell sensitive to HDAC6 inhibition has not been clearly defined [91,92,93]. Finding the right disease characteristics that sensitise to HDAC6 inhibition will be key to its application in the clinic.

Selective HDAC6 inhibitors such as ricolinostat and citarinostat were initially developed by chemical modifications to the pan-HDAC pharmacophore, in which these inhibitors also consist of a CAP, ZGB, and linker regions [94]. In general, increased acetylation of HDAC6 substrates such as α-tubulin occurs in the presence of selective HDAC6 inhibitors and indicates their pharmacodynamic activity, whereas the acetylation of lysine residue 9 (K9) of histone 3 demonstrates inhibition of class I HDACs [42,95]. Several recently published studies recorded ricolinostat as causing nonselective inhibition of class I HDACs in acute myeloid leukemic (AML) and high-grade serous ovarian cancer [96]. Moreover, the phenotypic responses associated with ricolinostat in tumours have been reported at concentrations higher than the HDAC6-selective concentrations in which the reported toxicity of IC50 doses were found to be high enough to inhibit nuclear HDACs [96]. The biological toxic effects of most of the small molecule inhibitors of HDAC6 recorded in the literature were produced at nonselective concentrations [38,42,95] and several studies did not measure the effect of the HDAC6 inhibitor concentration on acetylated levels of histone 3 [54,95]. Therefore, many of the earlier studies of HDAC6 inhibition in cancer did not specify its specific mode of action. To address limitations affecting the selective targeting of existing HDAC6 inhibitor molecules, extensive research has focused on optimising structural modifications to the core HDAC-targeting pharmacophore as reviewed by Huang et al., 2024. Addressing the lack of efficacy of HDAC6 inhibition as a monotherapy led to the development of several dual targeting molecules [87]. Nonetheless, discerning which tumours to treat will inevitably help stratify patients best suited to HDAC6 inhibitory therapy and improve the success of this class of drugs in the clinic.

HDAC6 mRNA or protein expression alone cannot stratify patients for HDAC6 inhibitory therapy, suggesting other mechanisms in the regulation of HDAC6 are at play [44]. The development of an approach to predict breast cancer sensitivity to HDAC6 inhibition successfully stratified high- and low-HDAC6-score cancers based on sensitivity to HDAC6 inhibition. The HDAC6 score was developed based on the transcriptional footprint of HDAC6 activity, which was shown to be highly conserved among epithelial cancers. An integrated analysis of cancer patient datasets for HDAC6-activity-affected gene expression profiles indicated that the UPR is the primary pathway associated with HDAC6 activity in cancer [97]. As the catalytic activity of HDAC6 is modulated by protein–protein interactions and post-translational modifications that may also allosterically control the catalytic domains of HDAC6 [14], the inhibition of HDAC6 in cancer with small molecule inhibitors requires consideration of the function it performs in the cancerous environment and if its expression in a tumour correlates with its deacetylase activity. Moreover, understanding the activation state of both HDAC6’s enzymatic and nonenzymatic functions could further help in identifying the most effective strategy for targeting HDAC6 in cancer.

## 3. Regulation of HDAC6 Catalytic Activity by Phosphorylation, Acetylation, and Protein–Protein Interactions

### 3.1. Regulation of HDAC6 by Phosphorylation

**Figure 1:**
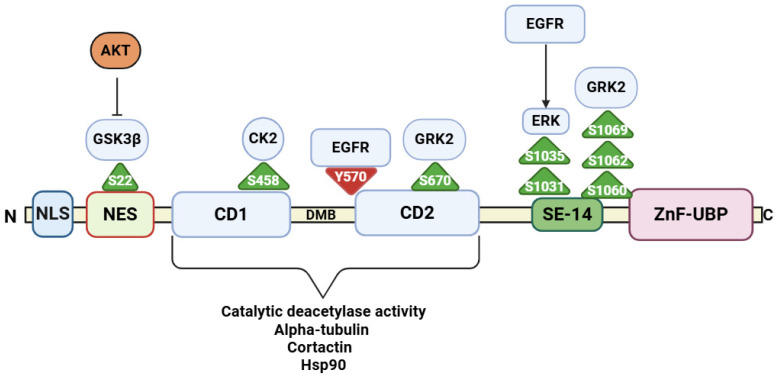
The major phosphorylation sites for serine (S) and tyrosine (Y) which modulate HDAC6’s activity are shown. Green and red triangles indicate increased or decreased catalytic activity. HDAC6 domains are labelled as catalytic domains (CD1 and CD2) and ubiquitin binding domain (UBP), nuclear export signals (NES), dynein motor binding region (DMB), a serine-glutamine-containing tetradecapeptide, cytoplasmic anchor (SE14), and nuclear localisation sequence (NLS). The protein kinases glycogen synthase 3β (GSK3β), casein kinase 2 (CK2), epidermal growth factor receptor kinase (EGFR), and G-protein coupled receptor kinase 2 (GRK2) responsible for each phosphorylation site are shown.

Phosphorylation is a post-translational modification performed by kinase enzymes, which regulate protein function and cellular processes by adding phosphate groups to specific amino acids in recipient proteins. The phosphorylation of HDAC6 can both enhance its deacetylation activity or reduce it (Figure 1). Although the regulation of HDAC6 by phosphorylation has not been extensively studied in the cancer context, many of the kinases which interact with HDAC6 are key mediators of oncogenic signalling. Furthermore, the phosphorylation of HDAC6 and subsequent modulation of acetylated-α-tubulin levels was found to have a direct impact on cell proliferation, migration, and motility [14]. Therefore, a detailed examination of these signalling events could enhance our understanding of HDAC6 inhibition in cancer. For example, HDAC6 is both a substrate of the epidermal growth factor receptor and phosphorylated downstream by mediators of EGFR signalling pathways [98,99]. Considering the constitutive activation of EGFR signalling in cancer drives cell proliferation, resistance to chemotherapy, and metastasis, HDAC6 responses may also contribute to EGFR responses in cancer. EGFR phosphorylates HDAC6 at tyrosine 570, which was shown to reduce HDAC6’s catalytic activity, resulting in increased α-tubulin acetylation [98]. Conversely, EGFR-mediated migration may occur because of HDAC6 phosphorylation at threonine 1031 and serine 1035, which increases its deacetylation activity towards α-tubulin. Serine 1035 serves as the primary site of extracellular regulated kinase (ERK)-mediated phosphorylation with phosphorylation at threonine 1031 dependent on the prior phosphorylation of serine 1035 [99]. Of note, HDAC6 is a significant regulator of EGFR signalling activity, in which the knockout of HDAC6 in lung cancer cells caused premature EGFR degradation and trafficking [13]. If constitutive EGFR activation phosphorylates HDAC6, this could impair HDAC6’s ability to regulate EGFR trafficking and degradation. Similarly, HDAC6-mediated deacetylation of ERK1 plays an important role in stimulating ERK1 activity in cell proliferation [100], highlighting a complex matrix of protein phosphorylation and deacetylation/acetylation events in growth factor signalling. Moreover, HDAC6 inhibition in the context of too much phosphorylation at tyrosine 570 is likely to be redundant and a futile therapy in this context.

Growth-factor-mediated activation of PI3K/AKT signalling is another critical signalling mechanism that becomes dysregulated in cancer cells. Phosphorylation of HDAC6 by GSK3β links HDAC6 with PI3K/AKT signalling; however, this response has only been described in neuronal cells in which GSK3β was shown to activate HDAC6 by phosphorylation on serine 22 [101,102]. Inhibition of GSK3β in hippocampal neuronal cells increased acetylated α-tubulin levels and enhanced mitochondrial trafficking due to reduced HDAC6 activity [101]. GSK3β is a constitutively active kinase, but its activity is inhibited when phosphorylated by the upstream signalling mediator AKT [103]. Therefore, in the presence of AKT, GSK3β constitutive activity towards HDAC6 would be diminished. On the other hand, HDAC6 has also been described to play a crucial role in regulating AKT dephosphorylation by forming a complex with protein phosphatase 1 (PP1). The inhibition of HDAC6 with the pan-HDAC inhibitor trichostatin A (TSA) is thought to prevent the formation of the HDAC6-PP1 complex in a dose-dependent manner, thereby promoting AKT dephosphorylation by PP1. Given the wide range of PP1-interacting substrates involved in growth factor signalling, HDAC6 may influence its own activity downstream of phosphorylation by regulating the access of PPI to its phosphosubstrates [104].

The atypical protein kinase C zeta (PKC ζ), which can act as either a tumour suppressor or promoter, depending on the cancer type, forms a complex with HDAC6 and was shown to phosphorylate serine and threonine residues in both the CD1 and CD2 catalytic domains of HDAC6 [105]. The formation of a complex between PKC ζ and HDAC6 is considered to be a predominant phosphorylation response of PKCζ and increasing levels of PKCζ in human embryonic kidney cells resulted in raised HDAC6 deacetylase activity and a dramatic reduction in acetylated α-tubulin levels. PKC ζ and HDAC6 inhibition were associated with comparable levels of acetylated-α-tubulin [105]. Several other kinases have been linked with decreased α-tubulin acetylation as a result of increased HDAC6 catalytic activity. The G-protein coupled receptor kinase-2 (GRK2) directly binds to the CD1 and CD2 domains of HDAC6, resulting in the phosphorylation of serine 670. The presence of GRK2–HDAC6 complexes at the leading edges of migrating HeLa cells is associated with increased GRK2-mediated cell motility and cancer metastasis [50,51]. The phosphorylation of serine residues 1060, 1062, and 1069, located between the CD2 catalytic domain and the ZnF-UBP domain, by GRK2 also regulates tubulin deacetylase activity. This interaction has been identified as an allosteric modification of HDAC6 by GRK2 phosphorylation that only affects its deacetylase activity toward specific substrates, such as tubulin. Conversely, modulation of GRK2 levels does not impact cortactin acetylation in cells and demonstrates that the post-translational modification of HDAC6 also modulates HDAC6 substrate specificity [51].

The phosphorylation of HDAC6 has been linked to the acetylated levels of other HDAC6 substrates. For example, PKCα was shown to phosphorylate and activate HDAC6 deacetylase activity towards β-catenin in viral immunity, although the exact phospho-sites in HDAC6 have not yet been described [106]. Considering that HDAC6 deacetylates β-catenin at lysine 49, a site frequently mutated in anaplastic thyroid cancer, the regulation of HDAC6 by PKCα may also influence β-catenin responses in cancer cells [26]. For example, in colon cancer cells, increased epidermal growth factor (EGF) signalling resulted in increased β-catenin acetylation, which was reduced in the presence of HDAC6 inhibition. HDAC6 inhibition prevented β-catenin phosphorylation, nuclear translocation, and transcription of the c-myc oncogene. Phosphorylation of HDAC6 also effects the transport and clearance of misfolded proteins in which casein kinase 2 (CK2) was shown to phosphorylate HDAC6 on serine 458 in the dynein motor binding region of HDAC6. In response to cell stress, phosphorylated serine 458 increases HDAC6 deacetylase activity and the binding of HDAC6 and misfolded proteins to dynein motor proteins [107].

### 3.2. Regulation of HDAC6 by Acetylation

**Figure 2:**
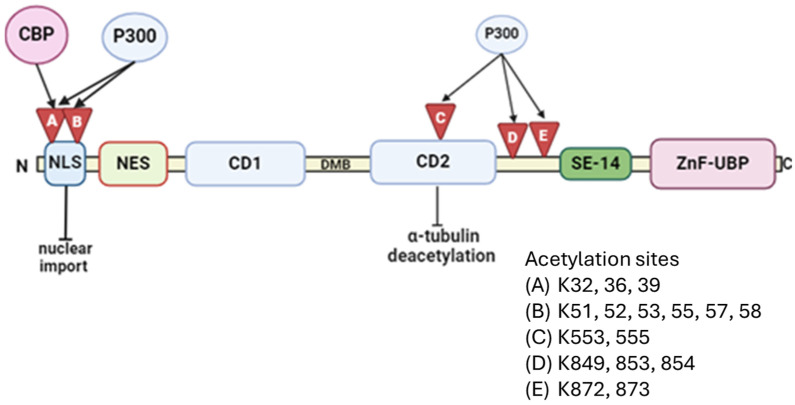
Schematic of the major acetylation cluster sites of HDAC6, which affects its catalytic activity. HDAC6 catalytic domains (CD1 and CD2) and ubiquitin binding domain (UBP), nuclear export signals (NES), a serine-glutamine-containing tetradecapeptide, cytoplasmic anchor (SE14), and nuclear localisation sequence (NLS). Histone acetyltransferases, CBP, and P300. Red triangles indicate that the presence of acetylation decreases the downstream function.

The acetylation of HDAC6 is less well described in the literature; however, existing studies indicate that acetylation at specific lysine clusters influences its activity. Protein acetyltransferases have been shown to acetylate lysine residues in the N-terminal nuclear localisation signal region, the CD2 domain, and the junction linking the CD2 domain to the SE region of HDAC6, thereby modulating its enzymatic activity and cellular localisation [108,109]. In HEK293 cells, the P300 transcriptional co-activator and HAT protein was identified as a key acetyltransferase that acetylates HDAC6. The acetylation of HADAC6 by P300 led to reduced HDAC6 deacetylase activity. Notably, P300 interacts with HDAC6’s catalytic domains with higher binding affinity compared to its C-terminal ZnF-UBP domain. This acetylation almost completely abolishes HDAC6’s ability to deacetylate α-tubulin, resulting in decreased cell motility [108,109]. The Creb binding protein (CBP) HAT was found to acetylate the N-terminal nuclear localisation signal region of HDAC6, inhibiting its nuclear import by disrupting interactions with nuclear import proteins. This cytoplasmic retention of acetylated HDAC6 consequently impairs its histone deacetylation activity [23,108]. In MCF-7 breast cancer cells, CBP-mediated acetylation and its effect on survivin deacetylation suggest that HDAC6’s subcellular localisation can be altered through interactions with acetyltransferase proteins and by regulating its binding with other protein partners. The acetylation of HDAC6 highlight the potential for the acetylation-dependent post-translational modifications of HDAC6 to impact its activity and cellular processes such as motility and nuclear signalling [23] (Figure 2).

### 3.3. Regulation of HDAC6 Function by Protein–Protein Interactions

**Figure 3:**
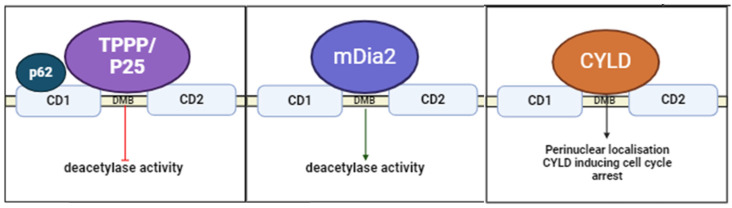
Schematic of major HDAC6–protein interactions, which regulate the catalytic function of HDAC6. The proteins tubulin polymerisation promoting protein (TPPP/P25) and cylindoramatosis (CYLD) interact with the dynein motor binding region (DMB). Sequestosome 1 (SQSTM1/p62) intern binds to the CD2 domain and decreases deacetylase activity along with TPPP/P25. CYLD binding to HDAC6 results in its localisation into the nucleus and causes cell cycle arrest by preventing the acetylation of α-tubulin.

The catalytic activity of HDAC6 is upregulated and downregulated by the proteins it binds, particularly in relation to its critical role in regulating microtubule dynamics (Figure 3, Table 3). Expression of the tubulin polymerisation promoting protein (TPPP/P25) is associated with increased tubulin acetylation, which results from decreased tubulin deacetylation. TPPP/P25 is an unstructured protein that was found to bind to and inhibit HDAC6 binding with tubulin with effects on microtubule network stability, bundling, and rearrangement that controls cell differentiation, maturation, and motility. It was found that the TPPP/P25 inhibitory effect of HDAC6 was compromised in the presence of increasing tubulin concentrations, suggesting that both tubulin and HDAC6 compete for the same specific binding domain of TPPP/P25. Therefore, in the presence of higher tubulin levels, the inhibitor effect of TPPP/P25 on HDAC6 is removed and HDAC6 activity increases. Inhibition of HDAC6 with TSA in the presence of TPPP/P25 further increased tubulin acetylation but was limited, suggesting that the active sites of the HDAC6 catalytic domains may become saturated [110]. The dynein light chain LC8 was also identified as a protein regulatory hub that mediates TPPP/P25 interaction with HDAC6. The association of TPPP/P25 with DYNLL/LC8 is attenuated in the presence of increasing tubulin concentrations, which may suggest competition of tubulin and DYNLL/LC8 for binding to TPPP/P25. Once TPPP/P25 binding to DYNLL/LC8 is decreased, it is available to bind to HDAC6 and, in the presence of DYNLL/LC8, tubulin acetylation decreases because of the inhibitory action of TPPP/P25 on HDAC6 [111]. LC8 alone has no effect on the degree of tubulin acetylation but, in the presence of TPPP/P25, tubulin acetylation decreased due to the impact of LC8 on the inhibitory potency of TPPP/P25 on HDAC6. Consequently, DYNLL/LC8 can directly bind to both TPPP/p25 and HDAC6, forming binary complexes, or it binds both proteins to form ternary complexes. These interactions are important for microtubule regulation [110,111].

The role of HDAC6 in aggresome formation depends on its interaction with microtubules, the modulation of tubulin by its catalytic activity, and also requires a series of protein–protein interactions. For example, sequestosome 1 (SQSTM1)/p62) is a scaffold protein involved in aggresomal formation and degradation. The downregulation of p62 resulted in the hyperactivation of HDAC6-mediated deacetylation of α-tubulin and cortactin. Higher HDAC6 activity increased the deacetylation of cortactin, leading to enhanced cortactin-dependent F-actin assembly with protein aggregates, but caused defective F-actin remodelling and autophagosome–lysosome clearance in the absence of p62 [112]. The binding region of p62 on HDAC6 is localised to the 429–824 residues, which span the entire CD2 domain. Therefore, P62 binds to HDAC6 CD2 domain, inhibiting its deacetylase activity [112]. HDAC6 interacting with p62 causes the association of HDAC6 with other interacting partners such as TRIM50 and parkin. The clearance of mitochondria by mitophagy occurs when p62 and HDAC6 are recruited to mitochondria that have been ubiquitinated by Parkin [113]. HDAC6 promotes the localisation of TRIM50 and E3-ubiquitin ligase to aggresomes, where it colocalizes with p62 and promotes the sequestration and clearance of ubiquitinated proteins [53]. Accordingly, the catalytic activity of HDAC6 in aggresomal degradation is regulated by the extent of HDAC6 protein interactions [112]. Likewise, the dynamic regulation of HDAC6 activity by protein interactions extends beyond aggresome biology, where research has described its broader role in cytoskeletal organisation. In osteoclast formation, which is a process that relies on the microtubule network, the modulation of acetylated tubulin levels by HDAC6 occurs downstream of another HDAC6 protein–protein interaction. The Rho effector protein mDia2 has been shown to bind to and enhance HDAC6 activity, leading to decreased tubulin acetylation. Regulation of tubulin acetylation by Rho is crucial for osteoclast maturation and demonstrates the interplay between microtubule dynamics and the actin cytoskeleton through HDAC6 modulation [114]. Of note, paxillin and F-actin-binding protein binds to HDAC6, which was found to have a significant role in cell migration and invasion [115]. HDAC6 activity is decreased in the presence of paxillin, resulting in increased microtubule acetylation. Furthermore, paxillin knockdown in breast cancer cells was shown to disrupt Golgi complex localisation and cell reorganisation as a result of an increase in HDAC6 activity in the absence of paxillin. HDAC6 inhibition reversed the cell responses caused by paxillin knockdown [115,116]. The regulation of HDAC6 by cylindromatosis (CYLD), which is a tumour suppressor protein mutated in benign skin tumours, shows that the regulation of HDAC6 activity by protein–protein interactions occurs in cancer. Binding between the catalytic domain of HDAC6 and the N-terminal region of CYLD reduces HDAC6 activity and results in increased tubulin acetylation, which facilitates the localisation of CYLD in the perinuclear region of dividing cells, causing cell cycle arrest [56].

### 3.4. Regulation of the Ubiquitin Binding Function of HDAC6

The presence of the ZnF-UBP domain in HDAC6 links the functions of HDAC6 to the ubiquitin system via conjugation with ubiquitin, polyubiquitin binding, and co-purification with deubiquitinating enzymes [117]. The ZnF-UBP domain of HDAC6 binds free unanchored ubiquitin and polyubiquintin proteins and is structurally different to other known ubiquitin binding domains [118]. While many ubiquitin-binding proteins facilitate proteasomal degradation by recognising lysine 48-linked polyubiquitin chains, HDAC6’s ubiquitin-binding domain binds lysine 63-linked polyubiquitin chains, assisting in the sequestration and transport of aggregated proteins [119]. The activity of the ZnF-UBP domain of HDAC6 is less active in full-length proteins compared to truncated C-terminal protein fragments, suggesting that HDAC6 protein conformation or catalytic domain activity regulates the ubiquitin-binding function of HDAC6 [120]. HDAC6 will specifically bind free ubiquitin chains but not ubiquitinated proteins and the ZnF-UBP in HDAC6 binds free ubiquitin with the highest known affinity [121]. Thus, binding between free ubiquitin and the HDAC6 ZnF-UBP domain would decrease its binding to polyubiquitinated proteins, where the recycling of free ubiquitin regulates the turnover of misfolded proteins [118]. The binding of HDAC6 to free ubiquitin is also associated with increased deacetylase activity towards cortactin and promotes F-actin polymerisation to facilitate proteasomal degradation and the clearance of proteins by autophagy [119]. The DBM region of HDAC6, which binds to dynein motors, promotes the loading of polyubiquitinylated misfolded proteins into dynein motors, forming complexes to facilitate its transport to the aggresomes in preparation for their degradation [52]. HDAC6 acts like a bridge between polyubiquitinylated misfolded proteins bounded to its ZnF-UBP domain and dynein motors bound to the DMB region [52]. HDAC6 was also found to be extensively colocalised with dynactin P150, which is also considered a component of dynein motor complexes [52]. The formation of HDAC6–dynein complexes increase as the misfolded protein levels increase, which suggests that the association of dynein motors to misfolded proteins is significantly affected by HDAC6 levels [52]. The deacetylase domains of HDAC6 are also required for aggresomal formation, suggesting that HDAC6 deacetylation of α-tubulin or other microtubules and actin-associated substrates modulate the binding of dynein motors to the DMB of HDAC6 with enhanced transport of misfolded proteins to the aggresomes [52]. Therefore, the deacetylation of microtubule-related proteins by HDAC6 may regulate the interactions between dynein, the DMB region of HDAC6, and the binding of polyubiquitinylated misfolded proteins to the HDAC6 ZnF-UBP domain [52]. Conversely, protein interactions between HDAC6 and P97/VCP modulate HDAC6 binding with polyubiquitinated proteins. P97 is an endoplasmic reticulum ATPase enzyme also called valosin-containing protein VCP or CDC48 in yeast [120]. Higher P97 expression compared to HDAC6 facilitates the removal of HDAC6 from polyubiquitinated proteins in a response, which favours the removal of cytotoxic proteins by proteasomal segregation and degradation, whereas higher HDAC6 levels facilitate removal of protein aggregates by the autophagic machinery [120]. Considering the central role of the ZnF-UBP to the functions of HDAC6, targeting the noncatalytic ZnF-UBP domain of HDAC6 offers an alternative approach to blocking substrate binding to HDAC6 [122]. Design of effective targeting of the ZnF-UBP in HDAC6 have already been reported [123]. Of note, the generation of a designed ankyrin repeat proteins (DARPins) prevents HDAC6 binding with ubiquitin and reduced influenza A virus (IAV) infection in a cell model of infection [124].

**Table 3:** Protein interactions involving HDAC6 and their functions.

Substrate	HDAC6-Related Interaction	Pathophysiology	Domains Involved	References
HDAC6 Ubiquitin Binding proteins				
Dynein	Binding to dynein motors Misfolded protein aggregation and degradation, cargos formation, and protein accumulation regulation	Neurodegenerative diseases, e.g., Parkinson’s disease, dementia with Lewy bodies DLB	DMB ZN-UBP CD1, CD2 required for functional activity	[14,52]
Ubiquitin	Protein ubiquitination, degradation, and endocytosis	Neurodegenerative Diseases	ZN-UBP	[14,52,120]
p97 VCP, p150Glued (dynactin]	AAA-ATPase in endoplasmic-reticulum-dependent proteasomal degradation	Neurodegenerative Diseases	Not identified yet	[14,120]
TRIM50 E3-Ubiquitin ligases	Recruitment of aggresomes and protein degradation	Neurodegenerative Disease, e.g., Alzheimer’s disease and Parkinson’s disease	Not identified yet	[14,53]
P62	The HDAC6 interacts with p62 by binding to its 164–225 residues Autophagic—lysosomal protein degradation regulation and promoting HDAC6 interaction with TRIM50	Neurodegenerative disease, e.g., Alzheimer’s disease and Parkinson’s disease	CD2 Mainly	[112]
HDAC6 Inhibitors				
P300 acetyltransferase	Interact and acetylate HDAC6, downregulating its catalytic activities	Gene transcriptional dysregulation	Mainly CD1 and CD2	[14,109]
TPPP/P25 (Tubulin Polymerisation-Promoting Protein/P25)	TPPP/P25 is α-tubulin acetylation modulator mediated by HDAC6 inhibition	TPPP has a role in the accumulation of α-Synuclein neurodegenerative disease such as Parkinson’s disease, Alzheimer’s disease, and Multiple System Atrophy	Not identified yet	[14,110,125,126]
DYNLL/LC8 (dynein light chain LC8)	Regulatory hub modulator enhances the inhibitory effect of TPPP/P25-HDAC6 interaction	---	Not identified yet	[111]
CYLD	The interaction of the first two N-terminal CAP-Gly domains with HDAC6 causes its inhibition, increasing α-tubulin acetylation, cell-cycle arrest, and cell proliferation inhibition	Skin tumour cells Melanoma	CD1 and CD2	[14,56]
Paxillin	Focal adhesion component inhibits HDAC6 deacetylase activity Mediated cell polarisation, directed migration and invasion	Tumour cell metastasis	Not identified yet	[116]
epidermal growth factor receptor kinase (EGFR-K)	HDAC6 phosphorylation at tyrosine 570 residue in CD2 domain, reducing HDAC6 deacetylase activity, regulation of EGFR trafficking, and degradation	----	CD1 and CD2	[13,26,98]
HDAC6 Activators				
Atypical Protein kinase C zeta (aPKC ζ)	HDAC6 phosphorylation of serein and threonine residues conserved in both catalytic domains CD1 and CD2 Increase HDAC6 tubulin deacetylase activity		Not identified yet	[105]
Calcium-activated protein kinase C α (PKCα)	PKCα induces HDAC6 deacetylase activity towards β-catenin and regulates its nuclear transcription and translocation	Viral infection and immunity	Not identified yet	[105]
protein casein kinase 2 CK2	HDAC6 phosphorylation of serein 458 residue in the dynein motor binding region, increasing the binding of HDAC6 and misfolded proteins to dynein motor protein in response to stress to modulate autophagic degradation	Neurodegenerative disorders, e.g., Alzheimer’s disease	Not identified yet	[107]
Extracellular Signal-regulated kinase ERK	HDAC6 phosphorylation of serine 1035 residue HDAC6-mediated cell motility occurs through EGFR–Ras–Raf–MEK–ERK signalling	----	Not identified yet	[99]
glycogen synthase 3β GSK3β	GSK3β phosphorylates HDAC6 serine 22 residue to enhance tubulin deacetylase activity Enhances neural mitochondrial trafficking	Neurodegenerative disorders	Not identified yet	[101]
G-protein coupled receptor kinase 2 GRK2	HDAC6 phosphorylation serine residues 1060, 1062, and 1069 in the region between the DD2 catalytic domain and the ZnF-UBP domain Improve HDAC6 tubulin deacetylase activity, enhancing cell migration	----	CD1 and CD2	[50]
mDia2 Mammalian diaphanous forming	Form a protein complex with HDAC6 to increase its deacetylation activity, cell mitosis, and maturation	Bone and calcium homeostasis regulation by osteoclast maturation and bone resorption dysfunction	CD1 and CD2	[14,114]
Other HDAC6 Protein complex				
PP1 Protein Phosphatase1	HDAC6 formed protein complex with PP1 The inhibition of HDAC6 causes the dissociation of this complex Increasing the AKT dephosphorylation, decreasing cell growth, and stimulating apoptosis	Anti-neoplastic effect Tumour cell growth and metastasis inhibition	Not identified yet	[14,104]

## 4. Conclusions

The specific application of HDAC6 inhibition in cancer remains uncertain, and there is considerable debate regarding the selectivity of HDAC6-targeting compounds, such as ricolinostat, which exhibit antiproliferative effects at concentrations exceeding the drug’s selective range. In addition, cancer cells often survive HDAC6 gene knockout, in contrast to the cytotoxic responses induced by catalytic domain inhibitors. Together, this suggests that these inhibitors may induce cell death through off-target interactions. Although further research is needed to clarify the selective nature of the HDAC6 inhibition, this may be less relevant if the off-target effects of drugs like ricolinostat are well tolerated by patients. Unlike pan-HDAC inhibitors, selective HDAC6 inhibitors have shown a favourable safety profile in clinical trials, with fewer dose-limiting toxicities, although, at higher concentrations, this is lost. Additionally, the ability of HDAC6 inhibitors to protect against chemotherapy-induced peripheral neuropathy further underscores their potential to improve patient quality of life irrespective of HDAC6-mediated anticancer activity. Nevertheless, the synergistic effects of combining HDAC6 inhibitors with other pathway inhibitors are well established and, if these therapies are to be administered successfully in the clinic, identifying which patients are most sensitive to HDAC6 inhibition will be valuable. As discussed in this review, HDAC6’s functional activity is complex, involving numerous post-translational modifications and protein–protein interactions. Research of HDAC6 inhibition rarely accounts for its activation state or non-deacetylase functions, despite these factors likely influencing the efficacy of inhibition. Additionally, phenotypic responses to HDAC6 inhibition differ from those following HDAC6 gene knockdown, further complicating our understanding of its role in cancer. Emerging research describing the HDAC6 regulon score introduced by Zeleke et al. attempts to correlate HDAC6 inhibition with the expression of genes regulated by its activity, offering valuable insights into the molecular context in which HDAC6 inhibition may be efficacious. Future research should also focus on both the refinement of existing compounds through medicinal chemistry and the exploration of alternative approaches, such as targeting HDAC6’s cancer-specific functions. Improving the inhibition of HDAC6 protein–protein interactions, for instance, could offer a more precise and effective therapeutic scalpel. In conclusion, correlating the cancer-specific expression of HDAC6 with its functional activity and activation state in tumours will be crucial for optimising HDAC6 inhibition as a therapeutic approach. This research will help to better identify the patient populations most likely to benefit, ultimately leading to more effective treatments and improved outcomes for patients.

## Figures and Tables

**Table 1:** Key HDAC6 substrates and their responses.

Substrate	HDAC6-Related Activity	Pathophysiology	Domains Involved	References
α-tubulin	Deacetylation of α-tubulin at Lys 40 residue, rearrangement of microtubule dynamics cell motility, migration and chemotaxis	Tumour cell metastasis Neurodegenerative disease (Parkinson’s disease, Alzheimer’s disease, and others)	CD1 and CD2 Both domains are required for complete deacetylase activity with the predominant impact of deacetylation by CD2 CD1 diminished the HDAC6 deacetylase activity	[3,13,14,15,16]
Cortactin	Deacetylation of 9 Lys residues present in the repeated region between its terminals Involved in cell migration and F-actin-based binding	Associated with actin-based cell motility disorders Tumour cell metastasis	CD1 and CD2 Both domains are required for the deacetylation interaction with cortactin	[8,14]
Heat Shock Protein HSP90	Deacetylation of HSP90 at Lys 294 Polyubiquitylation and proteasomal clearance of misfolded proteins	Neurodegenerative diseases Tumour cells metastasis	CD1, CD2, and ZN-UBP The catalytic domains alone have slight effect on HSP90 acetylation level and its chaperone function ability	[6,14,17]
Tau	Deacetylation of Tau on Lys 280 and 281 which is within the microtubule binding domain HDAC6 inhibition result in tau acetylation and increased its phosphorylation Ubiquitin binding tau degrading either proteolytic activity of HDAC6 or enhancing autophagy mechanisms	Tau aggregation and accumulation in neurodegenerative disease such as Parkinson’s disease and Alzheimer’s disease	CD1 and CD2 ZN-UBP	[14,18,19,20]
Ku70/Bax	Deacetylation of Ku70 at Lys **539**/542 residues Block the proapoptotic effect of Bax Anti-apoptosis pro-survival effect	In neuroblastoma (paediatric solid tumour): affect its survival but clinically not considered to be associated with aggressive tumour behaviour or poor patient outcome In pulmonary hypertension: enhances the proliferation, vascular remodelling, and survival of pulmonary arterial smooth muscle cells	Not identified yet	[14,21,22]
Survivin	Deacetylation at Lys 129 residue Antiapoptotic and survival function	Tumour cells, e.g., breast cancer	CD2	[14,23]
Peroxiredoxins (PrxI and PrxII)	Deacetylates PrxI at Lys 197 and PrxII at Lys 196 Oxidative stress induced cell death and redox system modulation	Tumour cells and neurodegenerative diseases Diabetes mellites induced myocardial infarction	CD2	[14,24,25]
β-catenin	Deacetylation of β-catenin at Lys 49 HDAC6-dependent epidermal growth factor induced nuclear β-catenin localisation and transcription Decreasing the expression of c-Myc oncogene	Tumour cell proliferation and metastasis	Not identified yet	[13,14,26]
Protein arginine methyl transferase 5 PRTM50	HDAC6 deacetylation of PRTM50 reduces its methyltransferase activity involved in proliferation and cellular response to stress and DNA damage.	---	CD1 and CD2	[27]
Myosin Heavy chain MHY9	HDAC6 deacetylation of MHY9 reducing its actin binding affinity Cell adhesion and migration	Tumour cell metastasis	CD1 and CD2	[12]
Hsc70/DNAJA1	HDAC6 deacetylation enhances Hac70/DNAJA1 interaction and its role in cell survival in response to cellular stress and hormonal receptor maturation	---	CD1 and CD2	[12]

**Table 2:** Cancer clinical trials involving selective HDAC6 inhibitors.

Compound ID	Condition or Disease (NCT Number)	Phase
ACY-1215 (Ricolinostat)	Multiple myeloma (NCT01997840)	I/II (completed and active)
	Relapsed/refractory multiple myeloma (NCT01323751)	I/II (active)
	Relapsed/refractory lymphoid malignancies (NCT02091063)	I/II (completed)
	Gynaecological cancer (NCT02661815)	I (active)
	Metastatic breast cancer (NCT02632071)	I (completed)
	Recurrent chronic lymphoid leukaemia (NCT02787369)	I (active)
	Cholangiocarcinoma (NCT02856568)	I (withdrawn)
ACY-241 (Citarinostat)	Malignant melanoma (NCT02935790)	I (completed)
	Multiple myelomas (NCT02400242)	I (active)
	Advanced solid tumours (NCT03008018)	I (active)
	Unresectable non-small cell lung cancer (NCT02635061)	I (active, not recruiting)
KA2507	Solid tumours (NCT03008018)	I (completed)
	Advanced Biliary tract cancer (NCT04186156)	II (Withdrawn)
